# Shifts in leaf N:P stoichiometry during rehabilitation in highly alkaline bauxite processing residue sand

**DOI:** 10.1038/srep14811

**Published:** 2015-10-07

**Authors:** Johnvie B. Goloran, Chengrong Chen, Ian R. Phillips, James J. Elser

**Affiliations:** 1 Environmental Futures Research Institute, Griffith School of Environment, Griffith University, Nathan, Qld 4111, Australia; 2 Environmental Research Department, Alcoa World Alumina Australia, Huntly Mine, P.O. Box 172, WA 6208, Australia; 3 School of Life Sciences, Arizona State University, Tempe, AZ 85287 USA

## Abstract

Large quantities of sodic and alkaline bauxite residue are produced globally as a by-product from alumina refineries. Ecological stoichiometry of key elements [nitrogen (N) and phosphorus (P)] plays a critical role in establishing vegetation cover in bauxite residue sand (BRS). Here we examined how changes in soil chemical properties over time in rehabilitated sodic and alkaline BRS affected leaf N to P stoichiometry of native species used for rehabilitation. Both Ca and soil pH influenced the shifts in leaf N:P ratios of the study species as supported by consistently significant positive relationships (*P* < 0.001) between these soil indices and leaf N:P ratios. Shifts from N to P limitation were evident for N-fixing species, while N limitation was consistently experienced by non-N-fixing plant species. In older rehabilitated BRS embankments, soil and plant indices (Ca, Na, pH, EC, ESP and leaf N:P ratios) tended to align with those of the natural ecosystem, suggesting improved rehabilitation performance. These findings highlight that leaf N:P stoichiometry can effectively provide a meaningful assessment on understanding nutrient limitation and productivity of native species used for vegetating highly sodic and alkaline BRS, and is a crucial indicator for assessing ecological rehabilitation performance.

Global production of bauxite residue during extraction of alumina from bauxite ore approximates 120 million tons every year^1^. Rehabilitation of bauxite residue sand (BRS) disposal areas is difficult and costly due to its inherent hostile characteristics–strong sodicity (ESP > 70%), high salinity (>30 dSm^−1^) and alkalinity (pH > 10) and nutrient scarcity and imbalance^2^,^3^. The establishment of a sustainable vegetation cover system in bauxite residue sand areas has been a key objective in rehabilitation activities. Gypsum addition has been known to effectively neutralize sodicity of bauxite residue areas^4^. Hence, it has become a vital part in rehabilitation approaches^5^,^6^, together with the addition of organic materials (e.g. wood chips for suppressing dust emission) and diammonium phosphate fertilizer as primary sources of nitrogen (N) and phosphorus (P) for vegetation establishment. Previous studies in BRS mainly concern (1) improvement of the physical and chemical properties via addition of organic and inorganic materials for supporting plant establishment and growth^7^,^8^,^9^,^10^, (2) water movement in the BRS profile^11^, (3) behavior of applied nutrients (e.g. DAP fertilizer)^12^, and (4) nutrient performance indices of both N and P^2^,^4^. By and large, these studies focus on improving the availability of macronutrients (e.g. N and P) to plants in the artificial environment of the bauxite residue sand disposal areas to sustain vegetation growth, and thus, improve ecological rehabilitation performance. However, understanding of shifts in soil N and P availability and the relative importance of N and P for vegetative growth and productivity during rehabilitation remain largely unknown.

The use of ecological stoichiometry such as leaf N:P ratio to characterize N and P limitation and saturation in relation to primary productivity at a given site has received much attention in the literature^13^,^14^. In particular, it has been suggested that leaf N:P ratios differ among and within plant species^15^ but that intraspecific variation is more substantial than interspecific variation for a particular species and can be used as an index of N or P limitation of plant production^15^,^16^. Despite potential variations among species, average thresholds for plant species at a given site have been suggested. For example, an average foliar N:P mass ratio for terrestrial plant, aquatic plant, and phytoplankton biomass is 12–13^17^. In a study of wetland plant species, leaf N:P was indicative of transition between N limitation and P limitation, with P limitation for ratios exceeding ~15 and N limitation at lower N:P^16^. Indeed, a variety of recent studies have indicated that leaf N:P ratio is a powerful tool to characterize nutrient stoichiometry in a wide range of settings such as in freshwater, marine and terrestrial environments^17^, in tropical trees^18^, semiarid grassland^19^, and in a ca. 500,000 year old dune chronosequence^20^. However, studies to examine the key factors that influence leaf N:P stoichiometry in strongly sodic and highly alkaline environments remain scarce, preventing application of ecological stoichiometry in restoration of bauxite residue sand disposal areas.

Generally, sodic-saline soils are characterized by having high pH (>8) due to excessive levels of exchangeable Na (NaCl and Na_2_CO_3_)^21^. These soil types inhibit plant productivity due to toxicity, nutritional imbalance and reduced osmotic potential^22^,^23^. Addition of Ca (gypsum) to sodic-saline and sodic soils has been reported to improve germination and biomass yields^24^, and to some extent improve N uptake, particularly for NO_3_^−^-N fed plants^25^. Moreover, the introduction of gypsum increased the Ca in soil and this in turn may affect nutrient availability. Grattan & Grieve (1992) reported that high salinity reduced foliar P concentrations in plants due to increased soil sorption of P by Ca given the low solubility of Ca-P minerals^22^. Furthermore, dominance of Na over Ca in soil can reduce grain yield and biomass production^21^. Likewise, solution culture studies have indicated that high amounts of Ca significantly stimulate shoot and root growth of *Oryza sativa L*.^26^. Meanwhile, it has been found that foliar CO_2_ fixation in *O. sativa L*. plants was reduced at increasing Ca levels in the nutrient solution^27^. High soil pH (>9) has also been reported to be a limiting factor for N and P availability and plant uptake in alkaline environment^5^,^6^. Despite the documented role of Ca, Na and pH in affecting nutrient availability and plant uptake, studies highlighting their role and function in plant nutrient (N and P) limitation and saturation under a sodic, saline and alkaline environment are scarce.

To address this limitation, here we investigated how sodicity, and alkalinity in rehabilitated bauxite residue sand disposal areas affect stoichiometric ratios of leaf N:P for the dominant native plant species (e.g*. Acacia rostellifera, Hardenbergia comptoniana and Eucalyptus gomphocephala*) grown in such environment. This study hypothesized that changes in soil chemical properties (sodicity and alkalinity) in rehabilitated BRS over time affect nutrient availability and plant nutrient utilization, thus influencing stoichiometric ratios of leaf N:P of vegetation growing in bauxite residue storage areas. Hence, the objectives of this study were to: (1) examine leaf N:P ratios of dominant native species growing in rehabilitated alkaline BRS under differing age of rehabilitation (2) understand nutrient limitation and productivity of native plant species currently used for vegetation of BRS, and (3) assess the suitability of leaf N:P stoichiometric ratios for characterising ecological rehabilitation performance in alkaline BRS.

## Results

### Concentrations and ratios of selected chemical properties of BRS and plant tissues

Chemical properties of BRS (TN, TP, N:P ratio, NO_3_^−^-N, AEM-P, Ca, Na, ECEC, ESP, EC and pH) and plant tissues (leaf N, leaf P, leaf N:P ratio,) revealed significant variations (Table 1). For example, there were significant (*P* < 0.05) variations found in the concentrations of total N, NO_3_^−^-N, total P, and extractable AEM-P including soil pH in BRS from sites where the study species were sampled and collected. In general, it appeared that availabilities of both N and P were significantly (*P* < 0.05) higher in BRS sampled beneath *H. comptoniana* and *A. rostellifera* than in BRS beneath *E. gomphocephala* (Table 1). Meanwhile, *H. comptoniana* significantly (*P* < 0.05) had the highest leaf N and P contents compared with *A. rostellifera* and *E. gomphocephala*. The leaf N:P ratios in *H. comptoniana* and *A. rostellifera* (leguminous plants) were significantly (*P* < 0.05) higher than the ratios found in *E. gomphocephala*.

**Figure 1:**
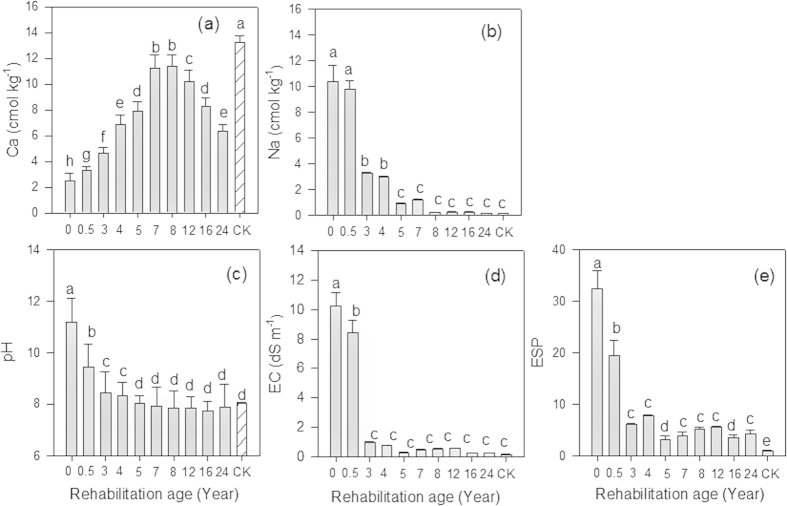
Effects of rehabilitation age on (**a**) Ca, (**b**) Na, (**c**) pH, (**d**) EC, and (**e**) ESP of BRS at Kwinana residue sand areas. Vertical bars (*n* = 3) with the same letter are not significantly different from one another at *P* < 0.05.

**Figure 2:**
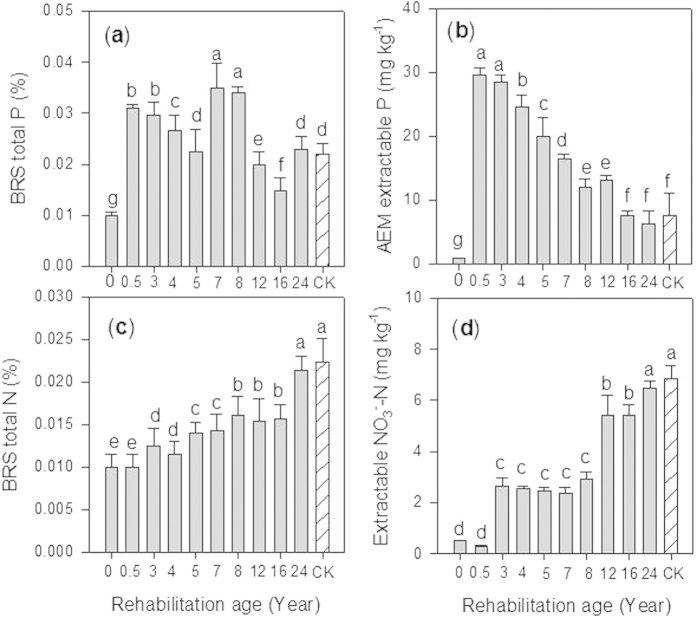
Effects of rehabilitation age on (**a**) total P (**b**) AEM extractable P (**c**) total N, and (**d**) extractable NO_3_^−^-N of BRS at Kwinana Residue Sand Areas. Vertical bars (*n* = 3) with the same letter are not significantly different from one another at *P* < 0.05.

Meanwhile, concentrations of Ca and Na in BRS revealed significant (*P* < 0.05) changes over time. Specifically, Ca tended to increase along with the age of rehabilitation while Na showed the opposite trend (Fig. 1a,b). Soil pH decreased from 11.20 (0 year) to 8.46 (3 years) and the electrical conductivity (EC) also showed a significant drop over the same period and continued to decline in old aged rehabilitated BRS (Fig. 1c,d). Similarly, the ESP declined significantly over time or with age of rehabilitated BRS (Fig. 1e). Total P and extractable AEM-P in BRS significantly (*P* < 0.05) declined while total N and extractable NO_3_^−^-N significantly (*P* < 0.05) increased with the age of rehabilitation (Fig. 2a–d). In addition, extractable NO_3_^−^-N revealed a consistent relationship with total N in BRS and BRS N:P mass ratio (Table 2).

### Leaf N:P ratios of native species and their relationships with soil indices in rehabilitated BRS

**Figure 3:**
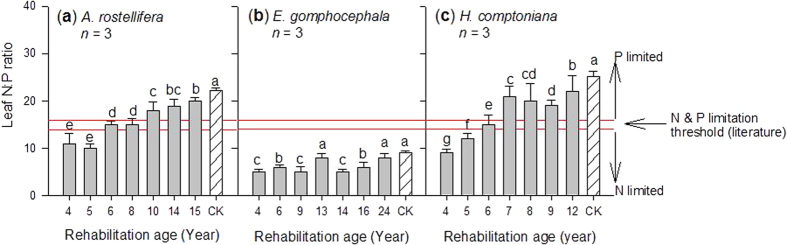
Leaf N:P mass ratios of various native species collected at Kwinana residue sand areas. N vs. P limitation threshold suggested by Gusewell & Koerselman (2002). Vertical bars (*n* = 3) with the same letter are not significantly different from one another at *P* < 0.05.

**Figure 4:**
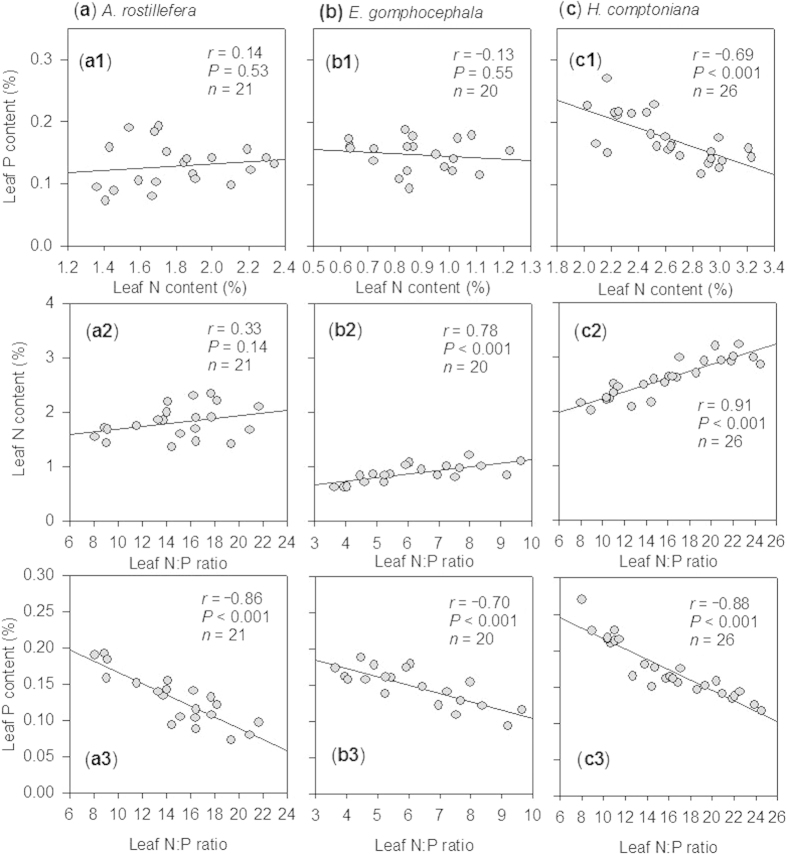
Relationships between leaf N, leaf P and leaf N:P mass ratio of *A. rostellifera* (a1–3), *E. gomphocephala* (b1–3) and *H. comptoniana* (c1–3) growing at Kwinana residue sand areas.

**Figure 5:**
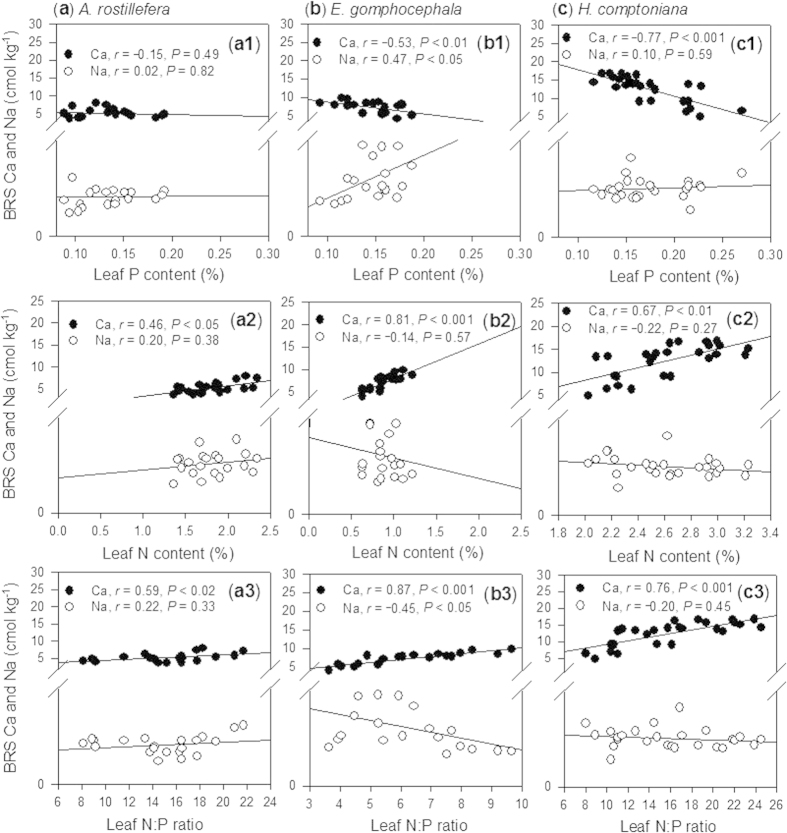
Relationships between soil indices (BRS Ca and Na), and plant indices (leaf N, leaf P and leaf N:P mass ratio) of *A. rostellifera* (a1–3, *n* = 21), *E. gomphocephala* (b1–3, *n* = 20) and *H. comptoniana* (c1–3, *n* = 26) growing at Kwinana residue sand areas.

Leaf N:P ratios for *A. rostellifera, E. gomphocephala and H. comptoniana* all increased with age of rehabilitated BRS (Fig. 3a–c). A comparable range of leaf N:P ratios was observed for both *A. rostellifera* (14.9, *n* = 21) and *H. comptoniana* (15.9, *n* = 26), while *E. gomphocephala* (6.22, *n* = 20) had the lowest N:P ratio among the study species (Table 1). Leaf P content had a significant (*P* < 0.001) and inverse relationship with leaf N:P ratio while leaf N content showed a positive relationship with leaf N:P ratios (Fig. 4a–c). Significant variations were observed in the relationships between leaf N:P ratios and other BRS chemical properties, such as pH, EC, and ESP (Table 2). Results also revealed highly significant (*P* < 0.001) relationships between leaf N:P ratios of the study species and soil indices such as Ca (Fig. 5a3–c3) and soil pH (Table 2). Both Ca and soil pH consistently obtained the highest correlation coefficients for their relationships with leaf N:P ratios of each plant species compared with the correlation coefficients for Na and other soil indices. In particular, leaf N:P ratio of *E. gomphocephala* had stronger relationships with pH, EC and ESP relative to *A. rostellifera, and H. comptoniana* (Table 2). While BRS extractable NO_3_^−^-N did not correlate with the leaf N:P ratios of *A. rostellifera* and *H. comptoniana*, it did have significant (*P* < 0.05) inverse relationships with the leaf N:P ratio of *E. gomphocephala* (Table 2). There was no significant relationship between leaf P or leaf N:P ratios and extractable AEM-P despite its (AEM-P) good correlations with BRS TP concentration or BRS N:P ratio (Table 2).

## Discussion

Concentrations of extractable N and extractable AEM-P in older rehabilitated BRS began to resemble the corresponding values in the reference sites (Fig. 2b–d). An increasing trend was observed for extractable N and for extractable AEM-P a decreasing trend with rehabilitation age (Fig. 2b–d). A tendency for increases in extractable N (NO_3_^−^-N) with BRS rehabilitation age has been reported previously^1^,^28^ as a result of subsequent litter decomposition, increased organic carbon, and release of organic N that together provide a favourable environment for microbial colonization (nitrifying bacteria) and N cycling. Conversely, the steady decrease in extractable AEM-P in older rehabilitated BRS resembles natural processes by which P is diminished in soil over time^20^,^29^. In particular, decreasing extractable P with rehabilitation age in the BRS can be attributed to increased uptake of P by plants^4^, leaching loss, complexation of P into low solubility compounds, and microbial immobilisation. Unlike N, sources of P in the bauxite residue sand disposal areas may entirely depend on applied inorganic P or the release of mineral P from decomposed organic materials, contrary to natural soil ecosystems that receive mineral P during weathering. The availability of N and P in BRS is crucial as this regulates ecosystem productivity^19^,^30^.

Freshly deposited BRS is extremely sodic due to processes involved during the extraction of alumina^1^,^2^. Thus, during early stages of BRS rehabilitation, gypsum is added to reduce sodicity. Our results showed that Na was dominant over Ca during the early stage (0 to 0.5 year) of rehabilitation (Fig. 1a,b). However, in 3- to 4-year old rehabilitated BRS, a significant shift was observed: Ca content increased dramatically while Na content declined significantly. This can be attributed to the effects of gypsum. As an ameliorant to BRS, it readily supplies Ca, which can effectively displace Na from the cation exchange sites^5^,^6^. Gypsum solubility and dissolution however, can be problematic. Previous work has described this problem despite attempts to use different types of water (seawater and triple-deionised water) to determine gypsum solubility in the freshly deposited BRS^4^. However, our results suggest the dissolution of gypsum in the field via natural leaching, as shown in the significant decline of Ca in the 12–24 years old rehabilitated BRS embankments (Fig. 1a). Despite the reduction, Ca remains dominant over Na in older rehabilitated BRS ecosystem. These however, only reflect the status of rehabilitated BRS at 0–10 cm and may not represent Ca and Na status at a depth >10 cm. Future studies on the belowground status of Ca and Na and other key nutrients in rehabilitated BRS should cover various depths (>10 cm) to greatly understand the implications on nutrient limitation and productivity.

High Ca contents with increasing age of rehabilitated BRS appear to regulate the availability of N and P as well as the uptake of these nutrients by plants in our study sites. Leaf N:P ratios for our study species had a strong positive relationship with BRS Ca concentration (Fig. 5a3–c3). Ca is well-known to markedly affect N and P availability in BRS, and thus the uptake of these nutrients by plants^2^,^31^. First, Ca reduces P availability in soil solution by adsorption^32^ or forming low solubility Ca-P minerals^22^. This mechanism inhibits P uptake by plants, resulting in low P concentration in plant biomass^33^. Indeed, such influences of Ca on soil P have been reported recently for ryegrass growing in BRS^4^. These conditions are more apparent in the present study where extractable AEM-P significantly declined with the age of BRS rehabilitation (Fig. 2b) while Ca concentrations increased for a certain period of time (0–8 years), and was consistently dominant over Na (Fig. 1a,b). Second, Ca may negatively affect N availability and particularly that of NH_4_^+^-N due to increased competition for limited exchange sites^31^. The dominance of Ca in the rehabilitated BRS may partially explain why we observed negligible amounts or below detection limit of NH_4_^+^-N (Table 1) but high concentrations of NO_3_^−^-N particularly in older rehabilitated BRS (Fig. 2d). This result is consistent with previous findings showing that NO_3_^−^-N was dominant over NH_4_^+^-N in BRS because the former was more stable than the latter in a highly alkaline environment^5^,^34^,^35^.

It has been reported that, in saline environments, NO_3_^−^-N performs better with increasing Ca supply (in soils) than NH_4_^+^-N for plant growth and biomass yield^25^. To some extent, the influence of Ca on N and P availability and plant uptake may explain the changes in leaf N:P ratios of the study species. Indeed, we found a stronger positive relationship between leaf N:P ratio and BRS Ca contents than between leaf N:P ratio and BRS Na contents for our study species (Fig. 5a3–c3). This relationship reflects a positive correlation between BRS Ca concentration and leaf N contents (Fig. 5a2–c2) but a negative correlation with leaf P content (Fig. 5a1–c1). The former correlation likely reflects the complementary effect (i.e. enhanced NO_3_^−^-N uptake by plants due to Ca protection of the nitrate transporter) between Ca and NO_3_^−^-N in the growth of plants^25^ because NO_3_^−^-N was the dominant source of N in the BRS that we sampled. This also corresponds to recent findings that leaf N contents of *A. rostellifera*, *E. gomphocephala,* and *H. comptoniana* were positively correlated with extractable NO_3_^−^-N in the Kwinana BRS disposal areas^2^. Moreover, BRS Ca concentration was negatively correlated with leaf P content for our study species, suggesting that increased Ca inhibited P uptake as discussed earlier. It is known however, that Australian native plants are inhibited by high concentrations of some soil nutrients (N and P), at which some are unable to survive^36^. Indeed, Lambers *et al.* reported that many of Australian native plant species in South-western Australia are extremely sensitive to soil P enrichment due to an inability to down-regulate phosphate-uptake capacity^37^. On the contrary, other native species tend to respond positively to P fertilization; this includes *E. gomphocephala,* which was observed to increase in abundance due to elevated soil P resulting either from increased fire frequency or fertilization^37^,^38^,^39^. Our study indicates that the dominance of Ca over Na in older rehabilitated BRS has differential influences on N and P availability and plant nutrient uptake, which are likely enhancing P limitation of plant production in the residue disposal areas.

Meanwhile, declining concentrations of Na with age of rehabilitated BRS (Fig. 1b) were consistently reflected in lower leaf Na contents compared with Ca contents across species (Table 1). We also found various relationships between leaf N:P ratios and leaf Na contents across species. For example, leaf Na showed a negative relationship with leaf N:P ratio for *A. rostellifera*, a positive relationship for *E. gomphocephala*, and no significant relationship for *H. comptoniana* (Fig. 5a3–c3). While these results suggest a variety of responses of plant species to the Na concentrations in rehabilitated BRS, they do support a view that changes in Ca and Na availability in rehabilitated BRS affect plant nutrient availability and utilization, thus affecting leaf N:P stoichiometry of the growing vegetation.

**Figure 6:**
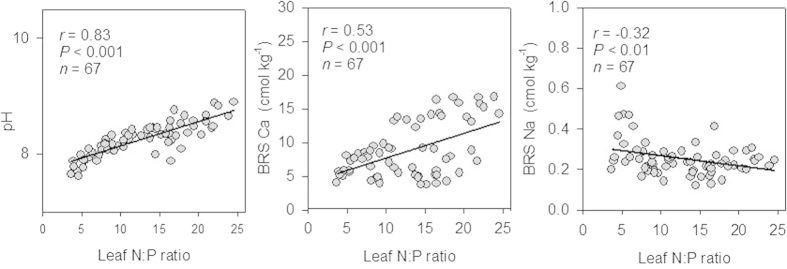
Relationships between leaf N:P mass ratios and Ca, Na and soil pH in rehabilitated bauxite residue sand areas at Kwinana, Western Australia.

Another factor that plays a critical role in driving the shifts in leaf N:P ratios of the study species in rehabilitated BRS is the soil pH. Soil pH was positively correlated with leaf N:P ratios of each study species (Table 2). By assessing the relationships (soil pH values and Ca vs leaf N:P ratios) on an intraspecific level (within plant species), the correlation coefficient favors BRS Ca over soil pH. When relationships however, are assessed based on an interspecific level (i.e. leaf N:P values of all study species were combined and plotted against soil pH and Ca), results would show the dominance of soil pH over BRS Ca in driving the shifts in leaf N:P ratios of the study plants (Fig. 6). Courtney and Harrington (2010) reported significant correlations of Ca and soil pH with P uptake by *Holcus lanatus* grown in residue embankment areas in Ireland^40^. These researchers found positive relationships for Ca (*r* = 0.415, *P* < 0.05) and negative relationship for soil pH (*r* = −0.379, *P* < 0.05) with P uptake or even with the biomass of *H. lanatus*. Our results revealed similar trend (negative relationship) with the soil pH. An opposite trend however, with BRS Ca, which may be due to the effect of differing age of rehabilitated BRS (i.e. varied effects of natural leaching by rainfall on gypsum dissolution) that we examined in this study. Moreover, significant effects of soil pH and Ca on N and P uptake and leaf N:P ratios of the dust control crop (*Lolium rigidum*) growing in various ages of rehabilitated alkaline BRS have been reported recently in a pot trial experiment^41^. Hence, results of this study show that soil pH along with Ca are key factors driving stoichiometric ratios of leaf N:P of plants growing in highly alkaline BRS areas, and are critical indicators influencing nutrient limitation and productivity in the alkaline environment of bauxite residue disposal areas.

Relationships between leaf N, leaf P and leaf N:P ratio or between leaf N and leaf P across species followed previously observed patterns (i.e. leaf N:P vs leaf P = negative correlation; leaf N:P vs leaf N = positive correlation), particularly those from experiments conducted at a field scale^16^. The only specific deviation significantly observed was that leaf N and leaf P for *H. comptoniana* were negatively correlated (Fig. 4c1). Among the study species, *H. comptoniana* had the highest leaf N content, ranging from 2.02 to 3.23% (Table 1); this high value could explain this pattern. In fact, variation of N has been suggested to be important in characterizing N:P ratios for woody plants, and plant species vary greatly in their individual responses to nutrient supply and in their internal nutrient translocation^15^.

While leaf N:P ratios of the study species demonstrated notable variations, all revealed an increasing trend with the age of rehabilitation (Fig. 3a–c). Variations among species may be due to differing capacities of each species to utilize the available nutrients^15^. Both *A. rostellifera* (N:P 8.10–21.7, *n* = 21) and *H. comptoniana* (N:P 8.02–24.5, *n* = 21) had comparable leaf N:P mass ratios across all rehabilitation ages and these were higher than those for *E. gomphocephala* (3.64–9.64, *n* = 20). It has been suggested that leaf N:P mass ratios in the range of 14–16 indicate N and P co-limitation, while values below and above this range indicate N limitation and P limitation, respectively^16^. Applying this threshold to characterize N and P limitations of the plant species growing in highly alkaline BRS indicates that both *A. rostellifera* and *H. comptoniana* are likely N limited during early stages of rehabilitation and then experience P limitation during later stages (Fig. 3a–c). In contrast, *E. gomphocephala* showed an inconsistent trend during early stages of rehabilitation and maintained leaf N:P ratios below the N limitation value despite a steady increase during the later stage (Fig. 3b), suggesting consistent N limitation throughout ecosystem rehabilitation. The increasing leaf N:P ratios across plant species was likely influenced by increasing N and decreasing P supply along with the age of BRS rehabilitation (Fig. 2b,d). At the field scale, this pattern of increasing N:P ratios can be expected due to atmospheric N deposition while P availability diminishes over time due to leaching and adsorption/precipitation^19^,^42^. At plant species level, these shifts could be due to the ability of plants to meet their demand for N via fixation, particularly for the *A. rostellifera* and *H. comptoniana*. However, substantial N fixation by these species requires that healthy root systems must first be established. Our data show that in 6-year rehabilitated BRS, leaf N:P ratios for both *A. rostellifera* and *H. comptoniana* shifted from values indicative of N limitation to values indicative of P limitation (Fig. 3a–c). This suggests that the N-fixing species may have established healthy root systems at this age. Similar dynamics have been reported by Dobrowolski *et al.* (2009), who showed that, despite restrictions on root growth by soil compaction in the BRS, healthy fine roots and root nodules of *H. comptoniana* were documented for in >5-year gypsum-irrigated BRS treatment site^43^. The ability of *A. rostellifera* and *H. comptoniana* to meet their own N requirements in the N-limited environment of the BRS likely resulted in increased leaf N:P ratios with rehabilitation age. This assumption is more simplified in the case of non-nitrogen fixing species such as *E. gomphocephala*. Despite somewhat increased leaf N:P ratios for the 14- to 24-year rehabilitated BRS (the oldest in the study), leaf N : P ratios were still low and indicative of N limitation. This indicates that *E. gomphocephala* experiences chronic N limitation in BRS and suggests that use of this species may not be sustainable. While it has often been thought that native species are efficient in utilizing available nutrients and are well-adapted to their nutrient-limited habitat^36^, this may not be applicable for all native species growing in the artificial conditions of BRS. Additional N input may be required to sustain production of *E. gomphocephala,* as this species is known to be more responsive to N than P fertilization^38^.

**Figure 7:**
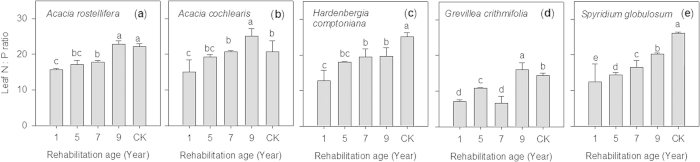
Leaf N:P mass ratios of various native species collected at Kwinana residue sand areas in various ages of rehabilitation. Vertical bars (*n* = 2) with the same letter are not significantly different from one another at *P* < 0.05.

Ecological rehabilitation performance of the study species can also be assessed when leaf N:P ratios of each species from the reference sites (i.e. samples obtained from the natural habitat) are used as an indicator of relative availability of N and P for native plants growing in the BRS. Aside from identifying which plant species are most suitable for sustainable ecological rehabilitation of the BRS disposal areas, our results also showed that, as rehabilitated BRS gets older (>16 years old), its nutrient dynamics mimic those seen in the natural ecosystems. This is seen in the observation that leaf N:P ratios of the studied species in the older rehabilitated BRS became similar to leaf N:P ratios of *A. rostellifera, H. comptoniana* and *E. gomphocephala* sampled from the reference sites (Fig. 3a–c). We observed similar trends for five native species (*Acacia cochlearis*, *Grevillea crithmifolia*, *Spyridium globulosum*, *A. rostellifera and H. comptoniana* sampled at Kwinana BRS disposal areas with rehabilitation age of 1, 5, 7 and 9 years old) when they are compared with respective leaf N:P ratios at the reference sites (Fig. 7a–e). These results suggest that, even though leaf N:P ratios of *E. gomphocephala* and *G. crithmifolia* are lower than the rest of the species, there are factors that result in increased leaf N:P ratios across species growing in the RSAs. Our data indicate that among these factors are Ca (i.e. dominance of Ca over Na) and soil pH (i.e. lower pH values as BRS gets older) that influenced N and P availability and utilization by plants as rehabilitated BRS ages, which we observed to resemble those in the natural ecosystem (Fig. 3a–c; Fig. 7a–e). Other chemical properties such as EC, ESP, extractable N, and extractable AEM-P may also contribute to leaf N:P variations, as these soil properties also shifted over time reaching values close to those obtained from the reference sites (Figs 1d,e and 2b,d).

Overall, we conclude that the variations in leaf N:P stoichiometry of the study species are influenced by the shifts from Na to Ca dominance and reduction in soil pH in rehabilitated BRS over time, which have major influence on soil N and P availability and plant nutrient uptake. Older rehabilitated BRS embankments mimic chemical characteristics and nutrient limitation of both soil and plants in the adjacent natural ecosystem, suggesting improved rehabilitation performance. These findings highlight that leaf N:P stoichiometry can effectively be used for understanding ecological nutrient limitation and plant productivity in rehabilitated highly alkaline bauxite residue areas. Future studies examining ecosystem productivity and nutrient dynamics at various depths or through the soil profile of BRS for improved nutrient management strategies and ecological rehabilitation performance are warranted.

## Materials and Methods

### Field survey

Since the 1960’s active aluminium production near the town of Kwinana (Western Australia; latitude 32°11′54.22″ South and longitude 115°49′31.93″) resulted in large bauxite residue areas that contain rehabilitated residue embankments of various ages. Rehabilitated BRS embankments referred to in this study were gypsum amended. As part of Alcoa’s rehabilitation protocol, gypsum is incorporated (225 t ha^−1^) to approximately 1.5 m depth that took place following the construction of the embankments^3^,^5^. Inorganic fertilizer such as Diammonium phosphate (2.7 t ha^−1^) including other nutrients such as K, Mg and trace elements are also applied prior to seedling or planting to improve nutrient cycling^5^,^6^.

Vegetation of native plant species in rehabilitated embankments was mostly thriving at 4 years old and above. Hence, collection of plants and BRS samples for this study were focused at >4 years of rehabilitated BRS embankments. Collection of BRS samples from less than 4 years old rehabilitated BRS however, was also carried out to examine changes in the chemical properties of BRS at the early stage of rehabilitation. Plant species commonly found in all areas of rehabilitation such as *Acacia rostellifera*, *Eucalyptus gomphocephala*, and *Hardenbergia comptoniana* were targeted in the sampling program. Sampling of BRS and associated vegetation was carried out by delineating 2-m^2^ quadrats in three replicates per sampled site. Rehabilitated BRS was covered by wood mulch (3 to 5 cm depth), which was carefully removed prior to collection of BRS samples. First, BRS samples (5–6 cores) from each plot were taken randomly using an auger (6 cm in diameter) beneath sampled plants to a depth of 10 cm. These were bulked as a composite sample. One portion of the BRS was taken for immediate chemical extraction of extractable N, while the other portion was air-dried and sieved (<2 mm) prior to chemical analyses. Second, biomass from three dominant plant species (*A. rostellifera, E. gomphocephala*, and *H. comptoniana)* was collected. Other species that can be found in some areas of the embankments (1, 5, 7 9 years old), such as *Grevillea crithmifolia, Acacia cochlearis* and *Spyridium globulosum,* were also collected. Similarly, samples of soils and focal species were also obtained from the natural habitat as reference or control samples. Specifically, fully open green leaves were collected, stored in paper bags, and rinsed with deionized water and oven-dried at 60 °C. All samples (BRS and plants) were finely ground with a mill prior to analysis.

### Soil analyses

Soil electrical conductivity (EC) and pH were measured in a 1:5 water extract. Total N and total P in BRS were extracted by Kjeldahl digestion method. Extractable N (NH_4_^+^ and NO_3_^−^) in BRS was extracted by 2 M KCl. Both the total N Kjeldahl digests and 2 M KCl extractable N extracts were analyzed using a SmartChem®200 Discrete Analyzer, WESTCO Scientific Instruments Incorporated. Extractable P in BRS was extracted by Anion Exchange Membrane (AEM-P) as described in Goloran *et al.* (2014)^4^. Total P Kjeldahl digests of BRS and AEM-P extracts were determined according to Murphy & Riley (1986)^44^. Exchangeable bases such as Ca^2+^, Mg^2+^, Na^+^ and K^+^ were extracted using the Mehlich III method, and extracts were analysed using ICP-OES (Varian Vista Pro Spectrophotometer). The ECEC and Exchangeable Sodium Percentage (ESP) were calculated directly, based on the concentrations of exchangeable cations as described by Hazelton & Murphy (2007)^45^.

### Plant analyses

Total N was extracted by Kjeldahl digestion method and analysed using a SmartChem®200 Discrete Analyzer as described earlier. Total P, Ca and Na were extracted using nitric-perchloric acid and the extracts were analyzed using the same instrument used for the Mehlich III extracts.

### Statistical Analyses

A Pearson correlation analysis was carried out using STATISTIX 8.0. Data for the chemical properties of BRS such as total P, total N, N:P ratio, Ca, Na, ESP, pH, EC and rehabilitation age, together with leaf N, leaf P and leaf N:P ratio, leaf Ca and leaf Na were subjected to correlation analysis. Analysis was carried out for both combined (*n* = 67) and separate species: *A. rostellifera* (*n* = 21)*, E. gomphocephala* (*n* = 20), and *H. comptoniana* (*n* = 26). The same software package was used to test the statistical significance of the data (age of rehabilitated BRS vs BRS and plant chemical parameters) using Analysis of Variance (ANOVA), and the mean differences were calculated at 5% level of significance using Tukey HSD All-pairwise comparison test.

## Additional Information

**How to cite this article**: Goloran, J. B. *et al.* Shifts in leaf N:P stoichiometry during rehabilitation in highly alkaline bauxite processing residue sand. *Sci. Rep.* **5**, 14811; doi: 10.1038/srep14811 (2015).

## Figures and Tables

**Table 1:** Selected properties of plants and BRS in the study.

Parameters	Field Survey at Kwinana RSA								
	*Hardenbergia comptoniana*(n = 26)			*Eucalyptus gomphocephala*(n = 20)			*Acacia rostellifera*(n = 21)		
	Range	Mean	Se	Range	Mean	Se	Range	Mean	Se
*Plant data*									
Leaf N (%)	2.02–3.23	2.73a	0.33	0.63–1.22	0.88c	0.16	1.36–2.34	1.80b	0.30
Leaf P (%)	0.12–0.27	0.17a	0.03	0.09–0.18	0.14b	0.02	0.07–0.19	0.12c	0.03
Leaf N : P mass ratio	8.02–24.5	15.9a	4.92	3.64–9.64	6.22b	1.78	8.10–21.7	14.9a	3.90
Leaf Ca (%)	1.05–1.90	1.35b	0.24	0.18–0.49	0.35c	0.08	1.68–2.73	2.22a	0.30
Leaf Na (%)	0.05–0.22	0.10b	0.04	0.10–0.25	0.20a	0.04	0.08–0.18	0.11b	0.03
*Soil data*									
BRS TN (g kg^−1^)	0.06–0.28	0.14a	0.10	0.06–0.50	0.12b	0.05	0.06–0.20	0.12b	0.04
BRS TP (g kg^−1^)	0.08–0.55	0.20b	0.10	0.06–0.61	0.26a	0.15	0.08–0.40	0.20b	0.10
BRS N:P mass ratio	0.11–3.52	0.76b	0.64	0.11–4.46	0.92a	0.99	0.15–1.43	0.62c	0.41
NH_4_^+^-N	BDL	BDL		BDL	BDL		BDL	BDL	
NO_3_^−^-N (mg kg^−1^)	2.28–8.85	5.11a	1.76	1.46–4.67	2.36b	0.76	1.85–3.25	2.56b	0.37
AEM-P (mg kg^−1^)	2.68–19.4	7.42b	4.10	2.76–12.3	5.77c	2.52	10.6–40.6	24.8a	8.52
Ca (cmol kg^−1^)	4.86–16.7	12.4a	3.59	4.06–9.77	7.21b	1.58	3.69–8.83	5.37c	1.42
Na (cmol kg^−1^)	0.14–0.41	0.25b	0.05	0.16–0.61	0.30a	0.12	0.12–0.30	0.21c	0.05
ESP (cmol kg^−1^)	1.25–8.82	3.85c	2.18	1.76–7.37	4.02b	1.79	2.59–8.82	4.24a	1.64
ECEC (cmol kg^−1^)	1.16–17.3	9.11a	4.93	4.56–10.3	7.92b	1.57	1.16–12.4	5.89c	2.39
pH	8.12–8.89	8.43a	0.23	7.62– 8.20	7.93b	0.16	7.87–8.67	8.32a	0.20
EC (dSm^−1^)	0.10–0.32	0.20b	0.06	0.13–0.71	0.40a	0.17	0.11–0.28	0.18b	0.05
Rehab age (year)	4–12			4–24			4–15		

*TN* total nitrogen, *TP* total phosphorus, *ESP* exchangeable sodium percentage, *EC* electrical conductivity*, Se* standard error, *BRS* bauxite residue sand, *Rehab* rehabilitation, *BDL* below detection limit. Means in rows (across plant species) followed by the same letter are not significantly different from one another at *P* < 0.05.

**Table 2:** Correlation coefficients (*r*) between BRS and plant indices from three native plant species in Kwinana residue sand areas.

	Leaf N	Leaf P	Leaf N:P	BRS N	BRS P	BRS N:P	NO_3_^−^-N	AEM-P	ESP	pH	EC	Rehab Age
*A. rostellifera* (*n* = 21)												
Leaf N	1	0.14	0.33	0.16	−0.14	−0.13	0.45*	0.12	−0.20	0.53*	−0.47*	0.26
Leaf P		1	−0.86**	−0.02	−0.13	−0.07	0.17	−0.10	−0.34	−0.11	−0.03	−0.57
Leaf N:P			1	0.10	0.32	-0.07	0.04	0.28	0.38	0.44*	−0.24	0.69**
BRS N				1	−0.30	0.66**	0.58**	−0.25	0.20	−0.19	−0.02	0.02
BRS P					1	−0.80**	−0.26	0.94**	0.32	0.62**	−0.51*	0.49*
BRS N:P						1	0.43*	−0.77**	−0.05	−0.62**	0.50*	−0.29
NO_3_^−^-N							1	−0.23	−0.05	−0.03	−0.03	−0.19
AEM-P								1	0.33	0.71*	−0.59**	0.45*
ESP									1	0.08	−0.10	0.16
pH										1	−0.79**	0.53*
EC											1	−0.26
Rehab Age												1
*E. gomphocephala* (n = 20)												
Leaf N	1	−0.13	0.71**	−0.26	0.19	−0.46*	−0.50*	−0.42	−0.47*	0.75**	−0.78**	0.48*
Leaf P		1	−0.78**	0.25	−0.34	0.23	0.41	−0.10	0.58**	−0.47*	0.29	−0.32
Leaf N:P			1	−0.35	0.36	−0.45*	−0.61**	−0.21	−0.71**	0.79**	−0.69**	0.54*
BRS N				1	−0.33	0.93**	0.79*	0.53	−0.01	−0.10	0.31	0.08
BRS P					1	−0.46*	−0.53*	0.40	−0.05	0.18	−0.20	0.05
BRS N:P						1	0.81**	0.52*	0.05	−0.23	0.43	0.04
NO_3_^−^-N							1	−0.28	0.30	−0.44*	0.59**	−0.30
AEM-P								1	0.05	−0.16	0.32	0.04
ESP									1	−0.67**	0.36	−0.49*
pH										1	−0.69**	0.38
EC											1	−0.48*
Rehab Age												1
*H. comptoniana* (n = 26)												
Leaf N	1	−0.69**	0.88**	−0.10	−0.31	0.15	−0.15	0.04	0.10	0.62**	−0.42	0.44*
Leaf P		1	−0.92**	0.06	0.09	−0.09	0.19	−0.18	0.15	−0.71**	0.64**	−0.30
Leaf N:P			1	−0.11	−0.25	0.09	−0.16	0.07	−0.06	0.76**	−0.63**	0.39
BRS N				1	−0.07	0.65**	0.72**	0.27	0.04	−0.03	0.18	0.15
BRS P					1	-0.054	−0.25	0.46*	−0.57**	−0.34	−0.01	−0.28
BRS N:P						1	0.49**	0.31	0.49**	0.24	0.02	0.19
NO_3_^—^N							1	−0.06	0.17	−0.04	0.17	0.12
AEM-P								1	−0.−7	0.02	−0.08	−0.12
ESP									1	0.11	0.22	0.33
pH										1	−0.72	0.45*
EC											1	−0.36
Rehab Age												1

*N* nitrogen, *P* phosphorus, *ESP* exchangeable sodium percentage, *EC* electrical conductivity, *BRS* bauxite residue sand, *Rehab* rehabilitation, *AEM-P* anion exchange membrane extractable phosphorus.

* and ** indicate significant at *P* < 0.05 and 0.01, respectively.
